# Study on synergistic effects of curcumin and bixin against foodborne pathogens

**DOI:** 10.1002/fsn3.3926

**Published:** 2024-02-22

**Authors:** Fereshteh Hosseini, Mohammad Bagher Habibi Najafi, Abdul Rasool Oromiehie, Mehdi Nasiri Mahalati, Masoud Yavarmanesh

**Affiliations:** ^1^ Department of Food Additives, Food Science & Technology Research Institute Iranian Academic Center for Education, Culture & Research (ACECR) Khorasan Razavi Branch Iran; ^2^ Department of Food Science and Technology, Faculty of Agriculture Ferdowsi University of Mashhad Mashhad Iran; ^3^ Department of Plastic Iran Polymer and Petrochemical Institute (IPPI) Tehran Iran; ^4^ Department of Agronomy, Faculty of Agriculture Ferdowsi University of Mashhad Mashhad Iran

**Keywords:** bixin, curcumin, *E. coli*, *L. innocua*, *S. aureus*, synergism, synergistic activity

## Abstract

Various studies have shown that natural colorants, in addition to their coloring attributes, have valuable biological effects such as antioxidant, anti‐inflammation, and anticarcinogenic properties. Moreover, their use as a food colorant can restrict the potential disadvantages of synthetic additives and turn foods into functional products. In this study, in vitro antimicrobial activities of two natural colorants of bixin and curcumin against some important foodborne pathogens: *Staphylococcus aureus* (*S. aureus*), *Listeria innocua* (*L. innocua*), and *Escherichia coli* (*E. coli*) were investigated by disk diffusion method. Minimum inhibitory concentration and minimum bactericidal concentration values were determined by agar dilution and broth microdilution methods. The synergistic activity of the colorants against selected microorganisms was assayed by the checkerboard microdilution method. The results showed that the inhibitory effects of bixin against *S. aureus* were more pronounced than *E. coli* and *L. innocua*. The lowest concentration of curcumin (0.6 mg/mL) in the disk diffusion method was not inhibited by any tested bacteria. However, it was effective at the higher concentrations against three microorganisms, but its diameter of inhibition zones was lower than gentamicin in all concentrations. Synergetic effects were observed by curcumin and bixin combination against *S. aureus* (FICI ≤ 0.5), but they act as an antagonist against *E. coli* and *L. innocua*. The results of the synergy test were confirmed by the isobologram curves.

## Graphical abstract

This study investigates the antimicrobial properties of two natural food colorants, bixin and curcumin, against three critical foodborne pathogens: *Staphylococcus aureus*, *Listeria innocua*, and *Escherichia coli*. The study found that bixin had more pronounced inhibitory effects against *S. aureus*. However, curcumin was effective at higher concentrations against all three microorganisms but had lower inhibition zones than gentamicin. The combination of curcumin and bixin showed synergistic effects against *S. aureus* while acting as an antagonist against *E. coli* and *L. innocua*.

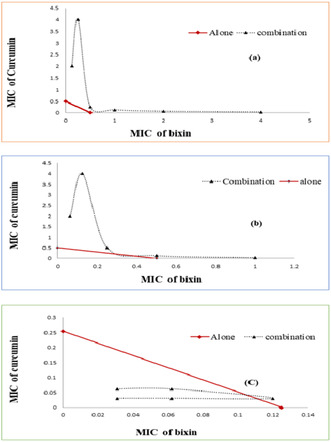

## 1 INTRODUCTION

Preservatives are often added to foods to prevent or reduce the growth of pathogens and spoilage microorganisms (Yu et al., 2021). However, certain synthetic food preservatives approved for consumption, such as nitrates and sodium sorbate, have been found to have toxic and mutagenic effects under certain circumstances (Carocho et al., 2014). As a result, consumers are increasingly demanding safe, efficient, and natural additives that have a broad‐spectrum capacity to inhibit the growth of foodborne pathogens (Gong et al., 2021). Colorants are important food additives to create an attractive and customer‐friendly appearance in food products and are generally used in various food products for instance confectionery, desserts, beverages, oils, pasta, etc. (Li et al., 2013). In recent years, there has been a growing demand for natural colors in food products, not only for their ability to add color but also for their positive functional effects. This trend is a result of the increasing public awareness of nutritional information and the desire for more natural ingredients in food (Nathan et al., 2019).

Curcumin is a fat‐soluble pigment with a low molecular weight that occurs naturally in turmeric (Adamczak et al., 2020). Its exceptional antioxidant properties, desirable coloring ability, and high safety have led to its approval as a food additive in multiple countries. Additionally, curcumin has various nutritional and pharmacological functions, such as reducing inflammation, fighting bacterial infections, and combating tumors. These properties have expanded its application to the field of biomedicine (Zhao et al., 2023). Curcumin is a type of polyphenol that can interact with proteins present in bacterial cell walls, leading to bacterial lysis. Its anti‐inflammatory properties are attributed to its ability to block cyclooxygenase‐2, lipoxygenase, and nitric oxide synthase. These enzymes play a crucial role in mediating inflammatory processes. Since inflammation is associated with tumor promotion, curcumin is believed to have chemopreventive effects. Its unique structure makes it an excellent antioxidant, mainly due to the H‐atom donation from the phenolic group (Boroumand et al., 2018; Jiang et al., 2019).

Annatto dye is a natural extract gained from seeds of *Bixa orellana* L. and has a wide application in food, cosmetic, medical, and hygienic industries. Different carotenoids have been identified in annatto seed, but its main pigments are bixin and norbixin. These pigments are extracted in different ways to obtain oil‐soluble extracts, oily suspensions, or water‐soluble extracts. Each kind of extract has its special applications (Chisté et al., 2011; Junqueira et al., 2018). Many studies have been performed to ensure the safety of annatto dye, and the results of these studies did not show any negative effects. Also, various antioxidant, anticancer, and antimicrobial properties are mentioned in the literature for bixin (Galindo‐Cuspinera et al., 2003; Siva et al., 2011; Tibodeau et al., 2010). Bixin possesses excellent antioxidant capabilities by capturing electrons, extinguishing singlet oxygen, deactivating photosensitizers' excited triplet state, and sweeping free radicals during their transition states. Bixin exhibits high anticancer potential and can reduce oxidative stress induced by lipopolysaccharide and high fat via the Nrf2 signaling pathway. It can also reduce proinflammatory cytokines by blocking the TLR4/NF‐B pathway. Carotenoids like bixin are capable of scavenging free oxygen radicals, which helps in curing (Ashraf et al., 2023).

When two functional compounds are used simultaneously, they can exaggerate or weaken each other's effects. A quantitative assessment is necessary to distinguish these cases from a simply additive action. The calculation is aided by a popular graph (isobologram) that provides a visual assessment of the interaction but also requires independent statistical analysis (Tallarida, 2001). It has been shown that bixin and curcumin have lethal or inhibitory effects on many bacteria. These two compounds are used to create color in food products, so they can also act as preservatives (Teow et al., 2016; Venugopalan & Giridhar, 2012; Yolmeh et al., 2014). There is no literature about their synergistic or antagonistic effects.

To introduce a natural safe preservative in food production against foodborne pathogens and to minimize the side effects of synthetic preservatives, this study aimed to evaluate the antibacterial activities of bixin and curcumin individually and in combination against some important foodborne pathogens *Staphylococcus aureus* (*S. aureus*), *Listeria innocua* (*L. innocua*), and *Escherichia coli* (*E.coli*).

## 2 MATERIALS

Turmeric rhizomes (*Curcuma longa* L.) were purchased from the local market in Mashhad, Iran. Annatto seeds (*Bixa orellana* L.) were sourced from Hyderabad, India. Bacterial strains used in antimicrobial analyses were *S. aureus* (ATCC25923), *L. innocua* (ATCC33090), and *E. coli* (ATCC25922) obtained from the American Type Culture Collection. Mueller–Hinton agar (MHA) and Mueller–Hinton broth (MHB), ethanol, n‐hexane, dimethyl sulfoxide (DMSO), the reagent of tri‐phenyl tetrazolium chloride were provided by Merck Millipore, and gentamicin (10 μg/dis) from Sigma‐Aldrich.

## 3 METHODS

### 3.1 Extraction of curcumin from turmeric

Turmeric rhizomes were milled and passed through a sieve with mesh 30. Curcumin was extracted from turmeric powder according to Apisariyakul et al. (1995) method with slight modification. Five hundred grams of dried turmeric powder was shaken continuously (750 rpm) with 600 mL of n‐hexane for 48 h. The extract was filtered to remove turmeric oil from the powder. Then, ethanol was used and after 24 h, the extract was filtered and concentrated in the rotatory evaporator under reduced pressure. Upon adding water to the concentrate, curcumin precipitated. The precipitate was filtered, dried in an oven at 50°C, and weighed. Finally, the precipitate was filtered and dried in the oven at 50°C. Curcumin powder was weighed and kept at 4°C before the test.

### 3.2 Extraction of bixin from annatto seeds

Bixin was extracted from annatto seeds by the procedure explained by Kovary et al. (2001) with some modifications. The 100‐g annatto seeds were extracted three times with 96% ethanol: H_2_O (90:10 v/v) for several hours with vigorous shaking. After filtration through gauze and then filter paper, the bixin remained on the filter paper was washed exhaustively by n‐hexane to remove all contaminants. After filtration, residual hexane was evaporated at 50°C, and bixin was obtained in a powdered form.

### 3.3 Preparation of inoculum

Active cultures of bacterial strains were prepared on MHA media. Bacterial cells from three to five similar colonies were transferred to sterile Ringer's solution using a sterile loop. A 0.5 McFarland standard was used for visual comparison to adjust the microbial suspension to a density equivalent to approximately 10^8^ CFU/mL. Further dilutions were performed using a sterile Ringer's solution.

### 3.4 Primary antibacterial activity assay

In order to determine the existence or lack of activity of curcumin and bixin against examined bacterial strains, the zone of inhibition test was used. A quantity of 100 mL of prepared inoculums (1 × 10^8^ CFU/mL) from each microorganism suspension was added to each plate and suspension was spread uniformly on the MHA plates by confluent swabbing of the surface. Blank sterile disks were soaked in 0.6, 1.2, 2.4, and 4.8 mg/mL solutions of curcumin and bixin in DMSO for 15 min. The fortified disks were transported to inoculated mediums and incubated for 24 h at 37°C. Gentamicin (10 μg/disk) as a positive control and disks soaked in DMSO as a negative control were also incubated. The antibacterial activity was evaluated by measuring the diameter zone of transparent inhibition against examined microorganisms with Caliper (in millimeters) and the inhibitory effects of curcumin and bixin were compared (Periago & Moezelaar, 2001).

### 3.5 Determination of the minimum inhibitory concentration (MIC) and minimum bactericidal concentrations (MBC)

As curcumin and bixin are naturally colored compounds, and to ensure the accuracy of the work, the determination of the MIC was performed using two methods: microdilution and agar dilution. MBC determination of curcumin and bixin was also carried out using broth microdilution method (Wiegand et al., 2008).

#### 3.5.1 MIC determination

In the agar dilution method, different concentrations of bixin and curcumin in appropriate solvents were prepared and added to plates. The sterile culture of MHA was then added to each plate and let to solidify. One hundred microliters of bacterial suspensions with a density of 1 × 10^4^ CFU/mL was inoculated to each plate and spread uniformly on the surface. Plates were incubated for 24 h at 37°C. The MICs were recorded as the lowest concentration of bixin and curcumin when no growth occurred on the plates.

In the broth microdilution method, stock solutions of bixin and curcumin were prepared in DMSO. Serial dilutions of bixin and curcumin using sterile MHB were made and poured into 96‐well plates (125 μL in each well). A quantity of 12.5 μL of freshly grown bacterial suspensions of 1 × 10^5^ CFU/mL was added to each well. After incubating at 37°C for 24 h, 25 μL of tetrazolium solution (5 mg/mL) was added to each well. The first well without bacterial growth (no red color was formed in the well) was reported as MIC.

#### 3.5.2 MBC determination

For determination of MBC, a sterile swab was soaked in the well with no turbidity (In broth microdilution method) and then transported to the MHA sterile mediums. After incubating for 24 h at 37°C, the lowest concentration related to the plates with no colony growth was reported as MBC.

### 3.6 Determination of in vitro synergic effects of curcumin and bixin

Synergic effects between bixin and curcumin against selected bacterial strains were tested by the checkerboard assay (Mackay et al., 2000). Serial dilutions of bixin and curcumin in twofold of their MICs were prepared in sterile MHB. Dilutions of bixin were distributed into the rows and dilutions of curcumin into the columns of 96‐well plates; therefore, in each well, the combination of the different concentrations of bixin and curcumin was poured. Each well was inoculated with 100 μL of bacterial suspension (5 × 10^5^ CFU/mL). After incubating at 37°C for 48 h, the synergistic effect was evaluated as a fractional inhibitory concentration index (FICI):

$$\text{FICI}={\mathrm{FIC}}_{\text{Curcumin}}+{\mathrm{FIC}}_{\text{Bixin}}$$

where FIC_Curcumin_ equals the MIC of curcumin in combination divided by the MIC of curcumin alone and FIC_Bixin_ equals the MIC of bixin in combination divided by the MIC of bixin alone.

The FICI was interpreted as follows:

FICI of ≤0:5 denotes synergism; 0.5 < FICI ≤4 shows indifference; FICI of >4 denotes antagonism (Karpanen et al., 2008).

### 3.7 Statistical analysis

For statistical analysis, a completely randomized design in factorial format was used to determine the antimicrobial activity of curcumin and bixin via Minitab version 21 software. All experiments were performed in four repetitions and differences between means were done using the Tukey's test at *α* = .05 level. EXCEL 2019 software was also used to draw graphs.

## 4 RESULTS AND DISCUSSION

### 4.1 Results of primary antibacterial activity assay

**FIGURE 1:**
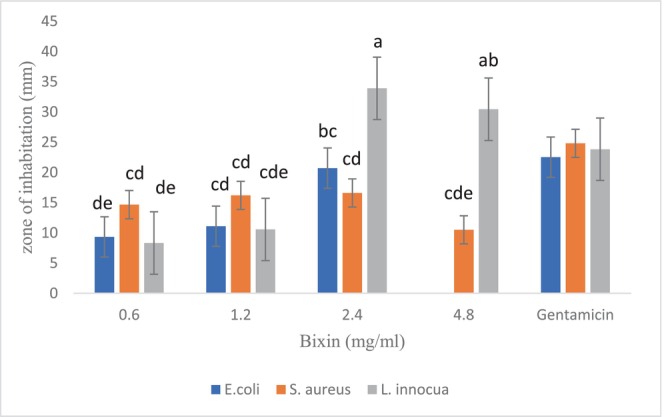
Diameter of inhibition zones of different concentrations of bixin against *Staphylococcus aureus*, *Escherichia coli*, and *Listeria innocua*. The presence of numbers with the same letters in each column indicates that there is no significant difference between the two samples (*p* < .05).

**FIGURE 2:**
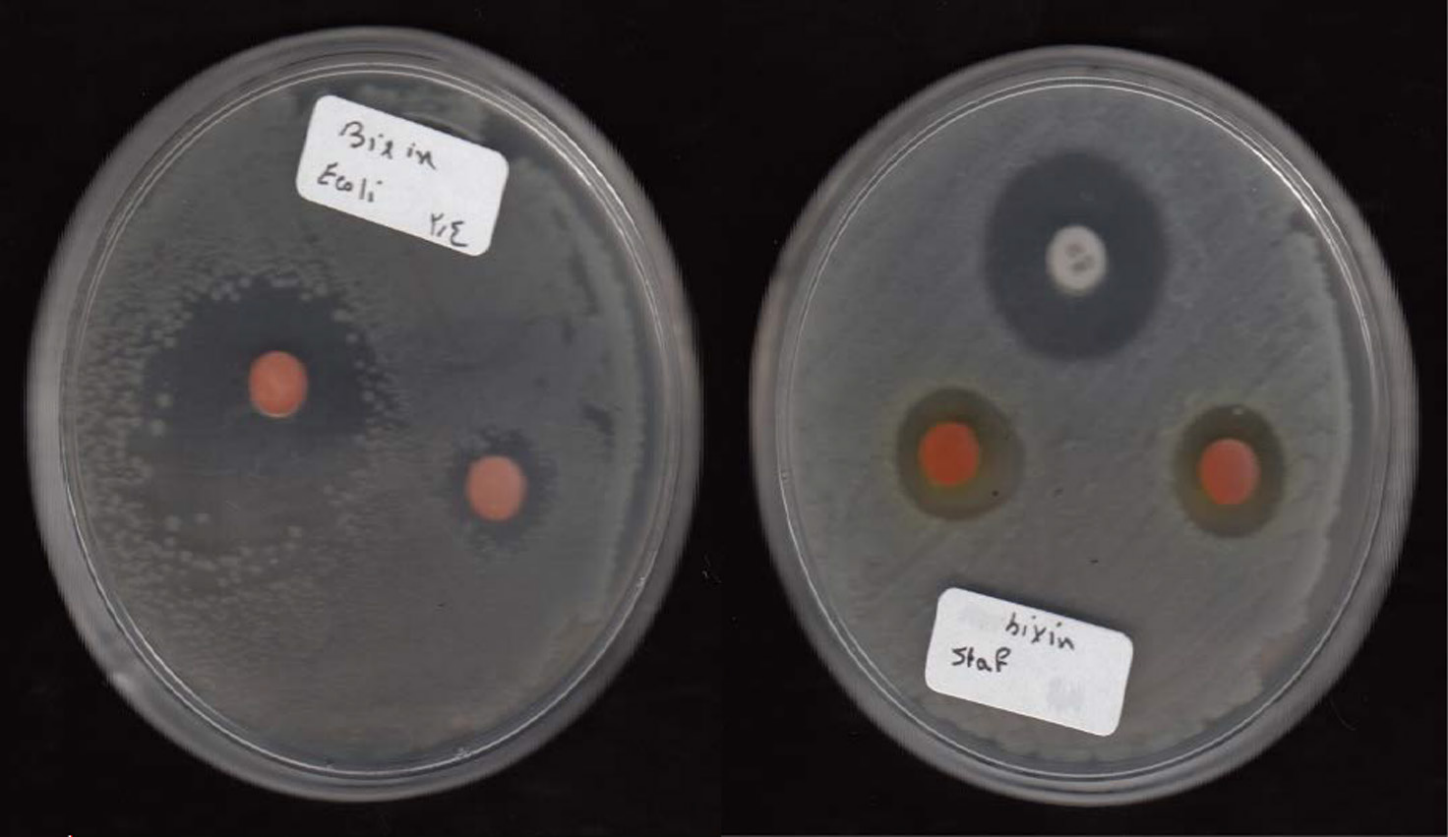
Inhibitory zones of bixin against selected bacteria.

Figure 1 summarizes the results of inhibition zones of different concentrations of bixin against three selected bacterial strains. Bixin exhibited broad‐spectrum inhibitory effects against all tested organisms (Figure 2). According to the results, it showed more inhibitory effects against *S. aureus* at the concentration of 0.6 and 1.2 mg/mL than other bacteria (*p* > .05). The concentration of 2.4 and 4.8 mg/mL of bixin formed a greater inhibitory zone against *L. innocua* compared to other bacteria (*p* < .05) and the standard antibiotic gentamicin disk (10 μg per disk). Also, bixin at the concentration of 4.8 mg/mL was a complete deterrent against *E. coli*, so no colony was grown on the plate.

The natural organic pigments of carotenoids can able to combat against pathogenic bacteria. Natividad and Rafael (2014) found that extracts from annatto, carrot, and tomato have antibacterial properties against *Staphylococcus aureus*. Among the three extracts, annatto extract showed the highest mean zone of inhibition at 9.17 mm, which was significantly higher than the other treatments but lower than the positive control, Streptomycin, with 34.67 mm. However, the extracts did not have any effect on the growth of *Escherichia coli*. It is noteworthy that annatto extract, which has the highest total carotenoid content, also exhibited the highest mean zone of inhibition against *S. aureus*. Therefore, the use of annatto extract can potentially prevent food spoilage caused by *S. aureus*. Fleischer et al. (2003) reported that the ethanol extract of annatto seeds (bixin) shows significant inhibitory activity against Gram‐positive and Gram‐negative bacteria such as *S. aureus* and some yeast‐like fungi such as *C. albicans*. They reported that the inhibitory diameter was equivalent to 20 mm and 22.5 mm for *S. aureus* and *E. coli*, respectively, at the concentration of 10 mg/mL bixin. Hajoori et al. (2013) also showed significant antibacterial effects of the annatto ethanolic extract. The researchers have introduced an inhibitory zone diameter equivalent to 11 mm for 10 mg/mL concentration of bixin against *E. coli*.

Comparing the results with other similar studies showed that the annatto extract displays dose‐dependent antimicrobial activity, a fact that was also emphasized by Hajoori et al. (2013). As reported in the literature, the amount of bacterial inoculum and concentrations of antimicrobial agents are effective in the results of inhibition tests (Bachir & Benali, 2012). The differences between the results of researchers can be also attributed to the variations of the antimicrobial compound concentrations in the initial plants and subsequently plant extracts. These variations depend on the genetic differences of plants or the environmental conditions in which the plant is grown (Silva et al., 2010). In the present study, n‐hexane is used to remove impurities besides ethanol and bixin was obtained with more purity, so it was more effective against microorganisms than the ethanolic extract of annatto seeds reported in other literature.

**FIGURE 3:**
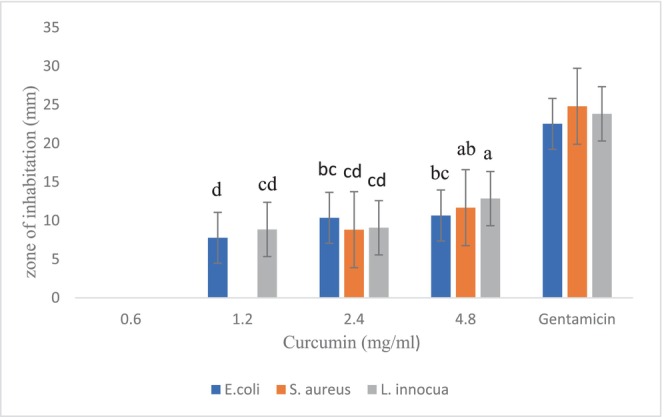
Diameter of inhibition zones of different concentrations of curcumin against *Staphylococcus aureus*, *Escherichia coli*, and *Listeria innocua*. The presence of numbers with the same letters in each column indicates that there is no significant difference between the two samples (*p* < .05).

The results of the antibacterial activity of curcumin are seen in Figure 3. According to the results, tested bacteria were not inhibited by the lowest concentration of curcumin (0.6 mg/mL); however, it was effective at the higher concentrations against three microorganisms. Curcumin exhibited more significantly inhibitory effects against *L. innocua* at the concentration of 4.8 mg/mL than *E. coli* (*p* < .05). However, the diameter of inhibition zones was lower than gentamicin in all concentrations. In comparing curcumin with bixin at the zone of inhibition test, curcumin showed less inhibitory effects against all microorganisms.

Phenolic compounds like curcumin are a diverse group of secondary metabolites found in edible plants. These compounds have been shown to exhibit antimicrobial properties by inactivating cellular enzymes. The rate of penetration of the substance into the cell and changes in membrane permeability play a crucial role in this process. By disrupting the cell membrane, phenolic compounds increase their permeability, leading to a loss of cellular integrity and eventually cell death. This mechanism is a major factor in the antimicrobial action of these compounds (Handayani et al., 2021).

Egan et al. (2004) reported that curcumin had significant inhibitory activity against *B. subtilis*, *E. coli*, and *S. aureus*. Wang et al. (2020) also conducted a study on the use of curcumin for quality and safety control in fresh millet noodle. The researchers found that curcumin exhibited effective antibacterial properties against *B*. *cereus* and *E*. *coli* in vitro. The results of the study also showed that curcumin was able to disrupt the bacterial membrane, leading to the formation of depressions and holes, cell lysis, and eventual death of the bacterial cells, as demonstrated by changes in cell membrane integrity, cell viability, and microstructure. Wang et al. (2009) determined the diameter of inhibition zones of encapsulated curcumin at the concentration of 200 mg/mL from 10.3 to 17.5 mm for *E. coli*, and *S. aureus*, respectively. Also, Taghinia et al. (2021) found that the film containing curcumin indicated clear microbial inhibition zones against both Gram‐positive and Gram‐negative bacteria. The diameters of inhibition zone were in the range of 18–20 mm for the tested bacteria.

### 4.2 Results of MIC and MBC

According to Table 1, MIC values of curcumin in the agar dilution method were larger than 0.35, 0.2, and 0.3 mg/mL against *E.coli*, *S. aureus*, and *L. innocua*, respectively. In this method, MIC values of bixin were larger than 0.4, 0.1, and 0.25 mg/mL against *E. coli*, *S. aureus*, and *L. innocua*, respectively.

**TABLE 1:** MIC of curcumin and bixin against selected microorganisms in agar dilution method.

Concentration (mg/mL)	*L. innocua*		*S. aureus*		*E. coli*	
	Bixin	Curcumin	Bixin	Curcumin	Bixin	Curcumin
0.05	+	+	+	+	+	+
0.1	+	+	+	+	+	+
0.15	+	+	−	+	+	+
0.2	+	+	−	+	+	+
0.25	+	+	−	−	+	+
0.3	−	+	−	−	+	+
0.35	−	−	−	−	+	+
0.4	−	−	−	−	+	−
0.45	−	−	−	−	−	−
0.5	−	−	−	−	−	−
0.55	−	−	−	−	−	−
0.6	−	−	−	−	−	−

*Note*: The sign of (+) shows the growth of the microorganism in the plate and the sign of (−) shows the lack of growth.

Antibacterial activity assessed by determination of MIC and MBC of curcumin and bixin against selected bacteria in broth microdilution method is shown in Table 2. As seen, obtained values in this method were in accordance with the agar dilution method.

**TABLE 2:** Results of MIC and MBC for bixin and curcumin against selected microorganisms broth microdilution methods.

Substance	MBC (mg/mL)			MIC (mg/mL)		
	Bacteria					
	*E. coli*	*L. innocua*	*S. aureus*	*E. coli*	*L. innocua*	*S. aureus*
Curcumin	0.5	0.5	0.25	2	2	0.5
Bixin	0.5	0.5	0.125	2	1	0.25

Results showed that the antimicrobial activity of bixin was more pronounced against Gram‐positive bacteria including *S. aureus* and *L. innocua* than Gram‐negative bacteria of *E. coli*. This commonly higher resistance among Gram‐negative bacteria has been attributed to an extra layer of lipopolysaccharide that covers their cell wall, which makes them more resistant to damage. On the other hand, Gram‐positive bacteria possess a cell wall as their outer layer, aside from those that have capsules (Bouyahya, 2016). Phenolic compounds and tetrapernoids such as tannins and carotenoids can disrupt the cell walls of bacteria when present in large amounts. However, the presence of extra defenses in Gram‐negative bacteria may hinder the efficacy of these compounds. This is why extracts of annatto do not exhibit antibacterial activity against *E. coli* (Natividad & Rafael, 2014).

To investigate the mechanism of the bixin effect on bacteria, Dinesh et al. (2011) conducted a study using SEM on various bacteria, including *S. aureus* treated with bixin. The researchers observed that the treated cells underwent degradation, aggregation, and disintegration. Indeed, Hajoori et al. (2013) found that the antimicrobial effects of annatto seeds' ethanolic extracts are attributed to alkaloids, flavonoids, and terpenoids.

Similarly, Shakeri et al. (2018) examined the potential of three carotenoids (annatto, paprika, and lutein) to determine whether they can be used as a substitute for chemical preservatives in food products. The results revealed that all the tested carotenoids, particularly annatto, exhibited antimicrobial effects, especially against Gram‐positive bacteria such as *Staphylococcus aureus*, *Staphylococcus epidermidis*, *Bacillus cereus*, *Bacillus subtilis*, *Listeria monocytogenes*, and *Streptococcus pyogenes*.

Curcumin exhibited stronger antimicrobial activity against Gram‐positive bacteria, as same as bixin. Curcumin (di‐feruloyl methane) is a polyphenol hydrophobic compound whose antimicrobial effects are attributed to its phenolic structure. It has also been reported that curcumin shows phototoxic effects in the presence of micromolar light. This compound has two conjugated electron systems in its structure. Conjugated bounds in various compositions can absorb visible light. They are responsible for the yellow color of the curcumin. The photo‐toxicity mechanism of curcumin may be due to hydrogen peroxide production and its toxic effects on microorganisms (Joe et al., 2004; Parvathy et al., 2009). Previous research has shown that curcumin can act as an antibacterial agent against *S. aureus* by inhibiting the bacterial surface protein sortase A, which prevents cell adhesion to fibronectin (Park et al., 2005). In addition, another study suggests that curcumin may exhibit antibacterial properties by binding to the cell wall of the bacterial cell, breaking it, and penetrating inside the cell, disrupting the structure of cell organelles (Bhawana et al., 2011).

In the same way as our study, the sugarcane bagasse extract, which contains high amounts of phenolic compounds, flavonoids, and phenolic acid compounds, showed antibacterial properties in vitro with MICs ranging from 0.1562 to 10 mg/mL against four tested bacteria. The MIC for *S. aureus* was 0.625 mg/mL, while for *E. coli* and *S. typhimurium*, it was 2.5 mg/mL. This result indicates that sugarcane bagasse extract has higher antibacterial activity against Gram‐positive bacteria than Gram‐negative ones. The bacteriostatic mechanism of the sugarcane bagasse extract is likely due to the toxicity of polyphenolic compounds to microorganisms, as suggested by Zhao et al. (2015). The study conducted by Gong et al. (2022) revealed that the MIC of cranberry anthocyanin against *S. aureus* strains was 5 mg/mL. Treatment with 2.0 MIC of cranberry anthocyanin for 0.5 h completely inhibited approximately 8 log CFU/mL of *S. aureus* strains. The treatment also resulted in a decrease in intracellular ATP and soluble protein levels, membrane structure damage, and cytoplasmic leakage.

### 4.3 Results of in vitro synergic effects of curcumin and bixin

Since both curcumin and bixin showed significant inhibitory effects against selected microorganisms, the interaction of these natural substances against three different strains of bacteria was evaluated by checkerboard microdilution assay, and FIC and FICI indexes were measured. Results are shown in Table 3.

**TABLE 3:** FIC and FICI of curcumin and bixin against selected microorganisms.

Substance	FICI			FIC		
	Bacteria					
	*E. coli*	*L. innocua*	*S. aureus*	*E. coli*	*L. innocua*	*S. aureus*
Curcumin	0.124	4	0.124	4.124	4.062	0.372
Bixin	4	0.062	0.248			

**FIGURE 4:**
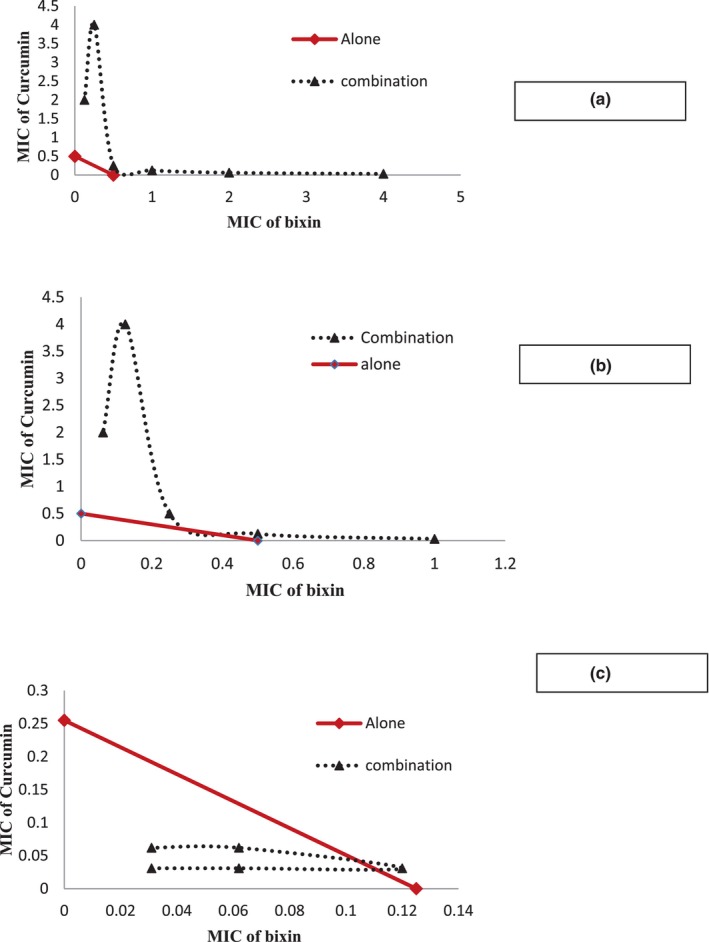
Isobolograms of combination effects for curcumin and bixin against *Escherichia coli* (a), *Listeria innocua* (b), and *Staphylococcus aureus* (c).

According to Table 3, synergetic effects were observed by curcumin and bixin combination against *S. aureus*, and fractional inhibitory concentration index (FICI) was less than 0.5, but the antagonistic effects were observed for two other microorganisms in the mixture of bixin and curcumin. These results confirmed with isobolograms of the combined effects of curcumin and bixin on the tested microorganisms. As seen in Figure 4, the natural color (bixin and curcumin) combination was more effective against *S. aureus*, and an increase in the combined bacterial inhibitory during the incubation time was determined (The combined effect curve is below the synergistic line). In the case of *E. coli* and *L. innocua*, the combined effect curve is above the synergistic line, which shows the antagonistic properties of the two compounds.

Synergism occurs when two compounds combine, creating a greater inhibitory effect than each compound alone (Kumar et al., 2014). In other studies, curcumin has been also shown to exhibit a synergistic antimicrobial effect with antibiotics and antifungals against several pathogens, including *S. aureus* (Teow et al., 2016). The results of Balan et al. (2016) suggested that the combination of curcumin, Manuka honey, and whey protein isolate could have synergistic and/or additive antimicrobial effects against various Gram‐positive and Gram‐negative bacterial strains. Also, the combination of other compounds has shown synergistic effects against *S. aureus*. Shi et al. (2017) demonstrated that the combination of nisin and Cinnamaldehyde exhibited effective synergistic antibacterial activities against *S. aureus*, which increased the efficiency of each compound alone. The compounds caused changes in the external structures of *S. aureus*, leading to deterioration of the cell wall and extensive cell lysis. Both nisin and cinnamaldehyde were found to damage the cell membrane and cell wall individually and also showed synergistic interaction based on the data from the membrane‐damaging effect assay and SEM assay.

## 5 CONCLUSION

This study confirmed the antimicrobial potential of bixin and curcumin to eradicate the bacterial infections of *S. aureus*, *E. coli*, and *L. innocua*, respectively. The combination of antimicrobial agents decreased the MIC of both agents and a synergistic effect was observed against *S. aureus*. However, the antagonistic properties of the combination were seen in the case of *E.coli* and *L. innocua*. These results were confirmed by the isobologram curves.

## AUTHOR CONTRIBUTIONS

**Fereshteh Hosseini:** Conceptualization (lead); data curation (lead); formal analysis (lead); investigation (equal); methodology (equal); project administration (lead); resources (equal); supervision (equal); validation (equal); visualization (equal); writing – original draft (lead); writing – review and editing (lead). **Mohammad Bagher Habibi Najafi:** Conceptualization (lead); data curation (lead); formal analysis (equal); methodology (equal); supervision (lead); writing – original draft (equal). **Abdul Rasool Oromiehie:** Conceptualization (equal); data curation (equal); formal analysis (equal); methodology (equal); supervision (equal). **Mehdi Nasiri Mahalati:** Formal analysis (equal); methodology (equal); supervision (equal). **Masoud Yavarmanesh:** Formal analysis (equal); methodology (equal); supervision (equal).

## FUNDING INFORMATION

This work was funded by the Department of Food Science and Technology Research Institute, Khorasan Razavi branch of the Academic Center for Education, Culture, and Research (ACECR) (11‐2067), located in ACECR Central building – University Campus, Azadi square, Mashhad, Iran.

## CONFLICT OF INTEREST STATEMENT

The authors declare no conflicts of interest.

## ETHICS STATEMENT

This study does not involve any human or animal testing.

## INFORMED CONSENT

Written informed consent was obtained from all study participants.

## Data Availability

Authors elect to not share data – research data are not shared.
